# Surgical Variations of the Modified Latarjet Procedure: A Literature Review and Video-Illustrated Surgical Technique

**DOI:** 10.7759/cureus.70221

**Published:** 2024-09-25

**Authors:** Khaled K AlAbbasi

**Affiliations:** 1 Orthopedic Surgery, King Fahad Medical City, Riyadh, SAU

**Keywords:** laterjet repair, recurrent dislocation shoulder, recurrent shoulder instability, shoulder instability, shoulder sport

## Abstract

The modified Latarjet procedure, otherwise known as the Walch-Boileau procedure, is a very successful management procedure for recurrent shoulder dislocation especially in patients with glenoid bone loss of less than 30 percent. Multiple variations of the surgical technique have been proposed over the years, some of which are still controversial. These variations include arthroscopic vs open technique, traditional versus congruent arc Latarjet procedure, subscapularis split versus L-shaped tenotomy, intra-articular versus extra-articular coracoid placement, capsular repair versus no repair, and the various modalities of coracoid fixation. The current evidence is based on a group of low-evidence heterogenous studies since some of these variations lack strong clinical comparative studies that control other variables. In this literature review, we present the evidence available for each of the above major variations. Furthermore, we present a video illustration of the surgical procedure done in our tertiary care center.

## Introduction and background

The Latarjet procedure is one of the most used procedures for the management of anterior glenohumeral instability. Although the utilization of the coracoid to prevent recurrent anterior shoulder dislocation was described in various ways earlier in the literature, the fixation of the coracoid to the anteroinferior portion of the glenoid by a screw to compensate for the bone loss in this area was first described by Latarjet in 1954 [1,2]. Latarjet attributed the success of his procedure to the bone block effect of the horizontal limb of the coracoid fixed to the anteroinferior margin of the glenoid. Patte expanded the explanation of the success of this procedure to what he called the “triple blocking effect”, which includes in addition to the bone block effect described earlier, the sling effect of the conjoint tendon on the inferior portion of the subscapularis tendon while the arm in abduction and external rotation and the statitic effect of repairing the capsule to the remnant of the coracoacromial ligament stump [3,4]. Although there is no agreement on which of these explanations contributes the most to the stabilization mechanism, the Latarjet procedure is considered one of the most successful surgeries used with a reported recurrence rate as low as 1% [1]. 

Since the first introduction of the Latarjet procedure, the surgical technique has undergone several modifications. In this paper, the major variations proposed in the literature will be discussed, including arthroscopic versus open surgery, traditional versus congruent-arc coracoid positioning, subscapularis split versus tenotomy, intra-articular versus extra-articular coracoid fixation, the management of the capsulolabral complex, and the methods of fixation of the coracoid to the glenoid. Furthermore, the surgical technique will be outlined, aided with a video illustration of these steps done in our tertiary care center based on the current evidence in the literature and the surgeon's preference. 

## Review

Open versus arthroscopic Laterjet procedure 

The concept and the technique of the arthroscopic Latarjet procedure were first introduced by Lafosse et al. in 2007 [5]. In their paper, they proposed several advantages over the open procedure including better visualization and exposure of the glenohumeral joint through using different portals, superiority in positioning of the coracoid graft, better soft tissue handling, decreasing the risk of scarring, reducing the risk of infection, faster rehabilitation, and better cosmesis [5]. To our knowledge, no randomized studies exist comparing the two techniques. The currently available evidence has failed to prove the superiority of the arthroscopic technique in any of the proposed advantages mentioned earlier. In fact, both techniques were comparatively highly successful in improving patient function with low complications and recurrence rates. Nevertheless, the arthroscopic technique is associated with a significant learning curve, longer operative time, and much higher cost [6-9]. 

Surgical Technique: Open Latarjet Procedure (Positioning, Approach)

**Figure 1:**
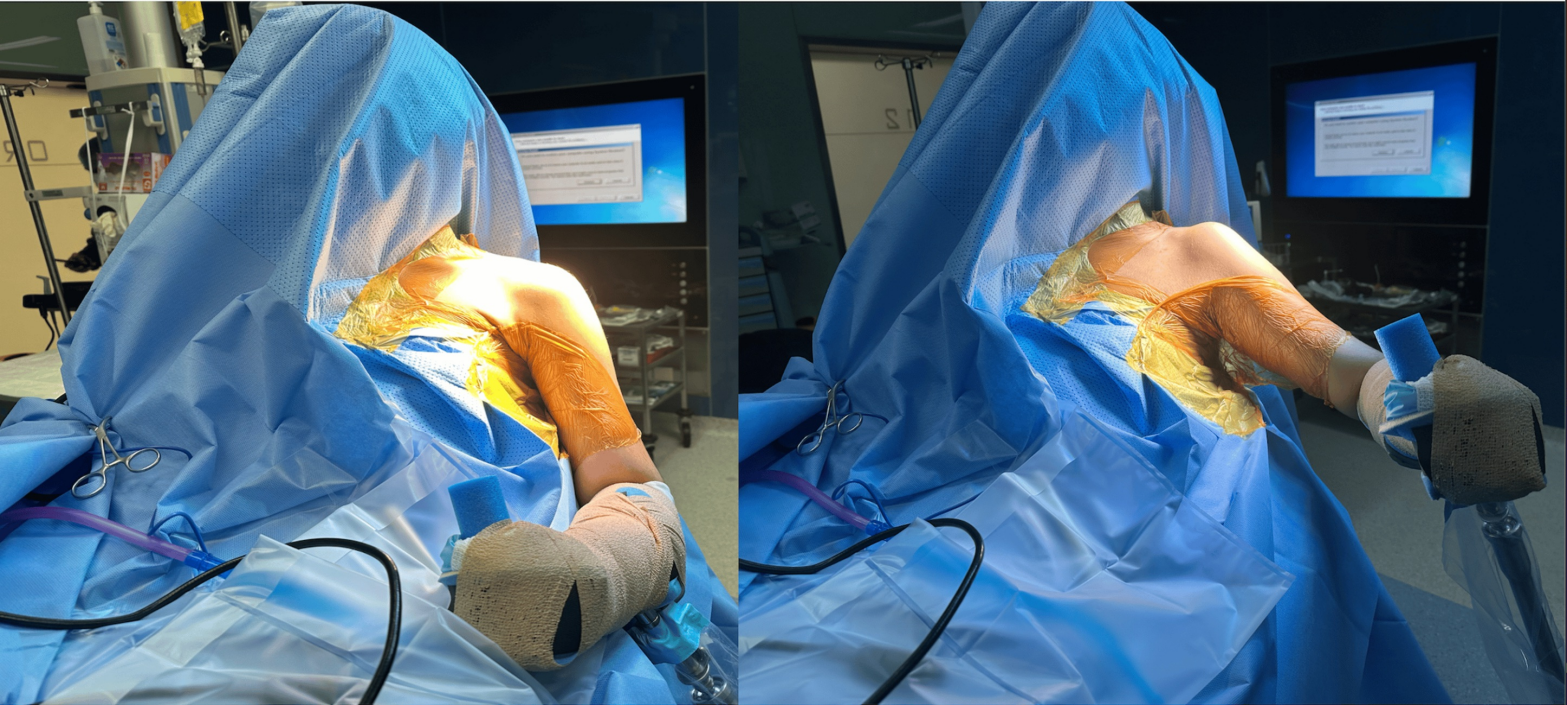
The patient is positioned in a beach position. The arm and the lateral half of the chest are prepped and wrapped. The forearm is connected to a mechanical arm-supporting device The picture was taken by the author after securing consent from the patient for taking images and videos for medical and research reasons.

The patient is placed in a beach chair position with a chair inclination of 45-60 degrees. The arm and the lateral half of the chest are prepped and draped in a fashion that allows free movement of the entire limb. If available, it is advisable to connect the forearm to a mechanical arm-supporting device to ensure steady holding of the shoulder and the arm in the desired position while performing the various surgical steps (Figure 1).

**Figure 2:**
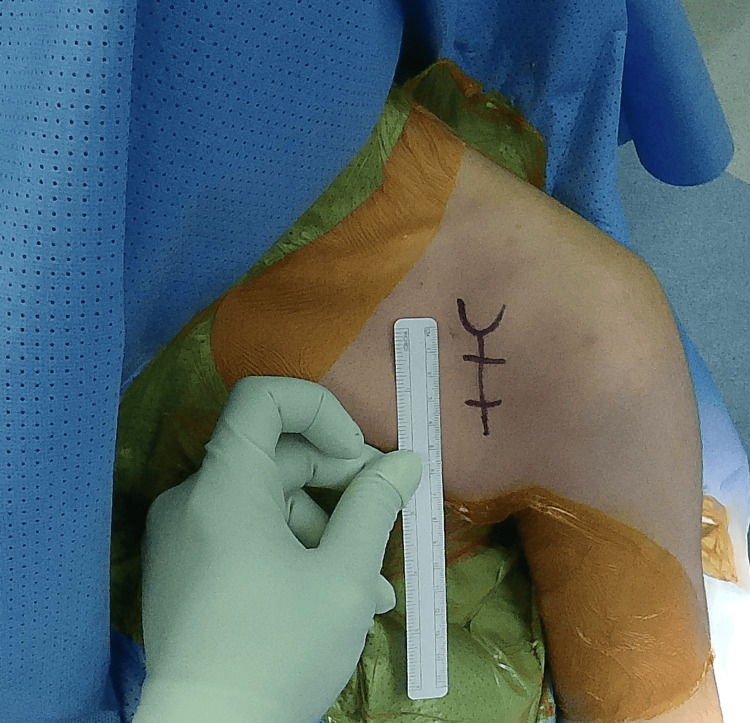
The incision is started at the level of the coracoid and directed toward the axilla The picture was taken by the author after securing consent from the patient for taking images and videos for medical and research reasons.

A 4-5 cm vertical incision is made starting at the level of the coracoid process and directed toward the axilla (Figure 2). After incising the skin, the subcutaneous layer superficial to the brachial and delto-pectoral fascia is raised as a full-thickness flap, both medially and laterally. The delto-pectoral interval is utilized by taking the cephalic vein medially or laterally and placing a self-retaining retractor to maintain the exposure. 

Traditional versus congruent-arc coracoid fixation

**Figure 3:**
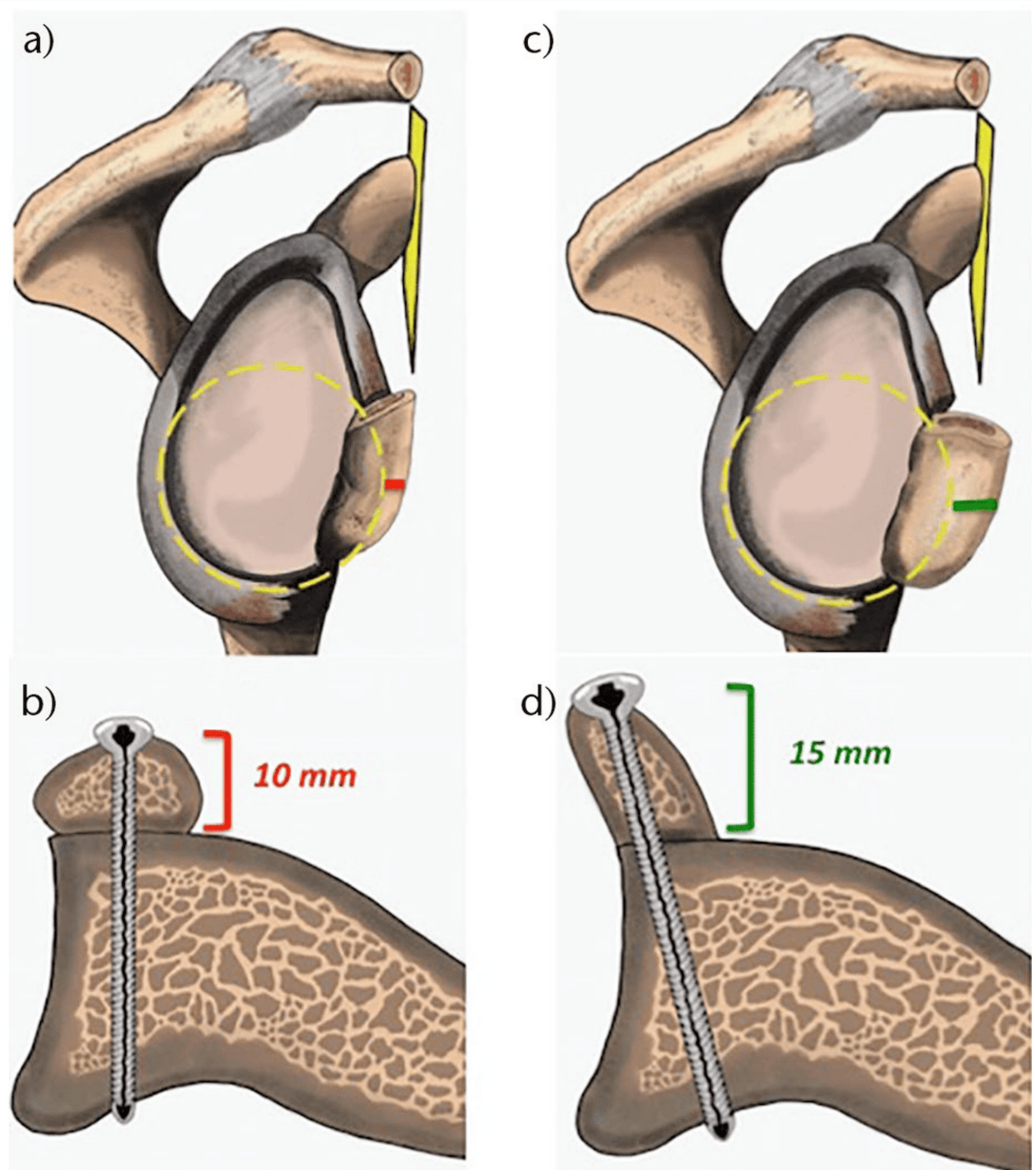
Traditional Latarjet (TL) technique versus congruent-arc Latarjet (CAL) technique. Sagittal view (a) and axial view (b) of a glenoid reconstructed with the traditional procedure. Sagittal view (c) and axial view (d) of a glenoid reconstructed with the CAL procedure The figure is taken with permission from [11].

To better understand the difference between the traditional Laterjet (TL) technique and the congruent-arc Latarjet (CAL) technique, it is important first to know the average coracoid dimensions. The coracoid bone is 15 mm in width (medial to lateral) and 10 mm in thickness (superior to inferior) [10]. In the TL technique, the inferior surface of the coracoid is fixed to the anterior glenoid neck, providing a 15 mm contact surface for bone consolidation and a 10 mm bone block for defect reconstruction. On the contrary, in the CAL technique, the coracoid is rotated 90 degrees, so the medial edge of the coracoid is fixed to the anterior glenoid neck providing a 10 mm contact surface for consolidation, while the inferior surface of the coracoid is in continuity with the glenoid articular surface, providing a 15 mm bone block for defect reconstruction (Figure 3) [11]. In their original paper proposing the CAL technique, Burkhart et al. proposed that the usage of the wider inferior surface of the coracoid for the reconstruction of the glenoid maximizes the bone block effect [12]. 

Multiple anatomical and biomechanical studies compared the potential superiority of the TL and the CAL techniques. Both techniques were found to provide adequate reconstruction of glenoid defects with less than 30-35% bone loss [10-14]. In a cadaveric study by Yamamoto et al., the sling effect of the conjoint tendon on the lower third of the subscapularis was found to be the main contributor to stability after Latarjet procedures, accounting for 51 to 62% of the stability in midrange of motion and 76 to 77% in the end range of motion, while the bone block effect contributes to 38 to 49% of stability in the midrange of motion and no contribution in end range of motion [15]. Given the low incidence of glenoid defects >35% in size and the superiority of the sling effect in providing stability, the bigger bone block effect provided by the CAL technique is not of clinical significance.

**Figure 4:**
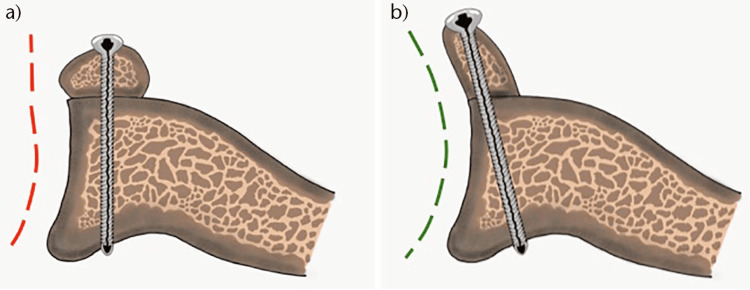
The radius of curvature of the reconstructed Glenoid after TL (a) versus CAL (b) techniques. The CAL technique provides a more anatomical radius of curvature and so less contact pressure in the glenohumeral joint The figure is taken with permission from [11]. TL: Traditional Latarjet; CAL: congruent-arc Latarjet

In addition to the bigger bone block effect, advocates of the CAL technique propose it as a better reconstruction option since the inferior concaved surface of the coracoid matches the concavity of the glenoid articular surface, thus providing more anatomical radius of curvature, and so decreases the contact pressure between the humeral head and the glenoid (Figure 4) [11,12,14,16]. Theoretically, this minimizes the risk of osteoarthritis associated with traditional Latarjet procedures [11,12]. Given the multiple other variables that may contribute to the risk of osteoarthritis and the lack of long-term studies on the risk of glenohumeral osteoarthritis after Latarjet performed with the CAL techniques, this proposal remains theoretical and need to be supported by long-term comparative studies that control the other variables. 

**Figure 5:**
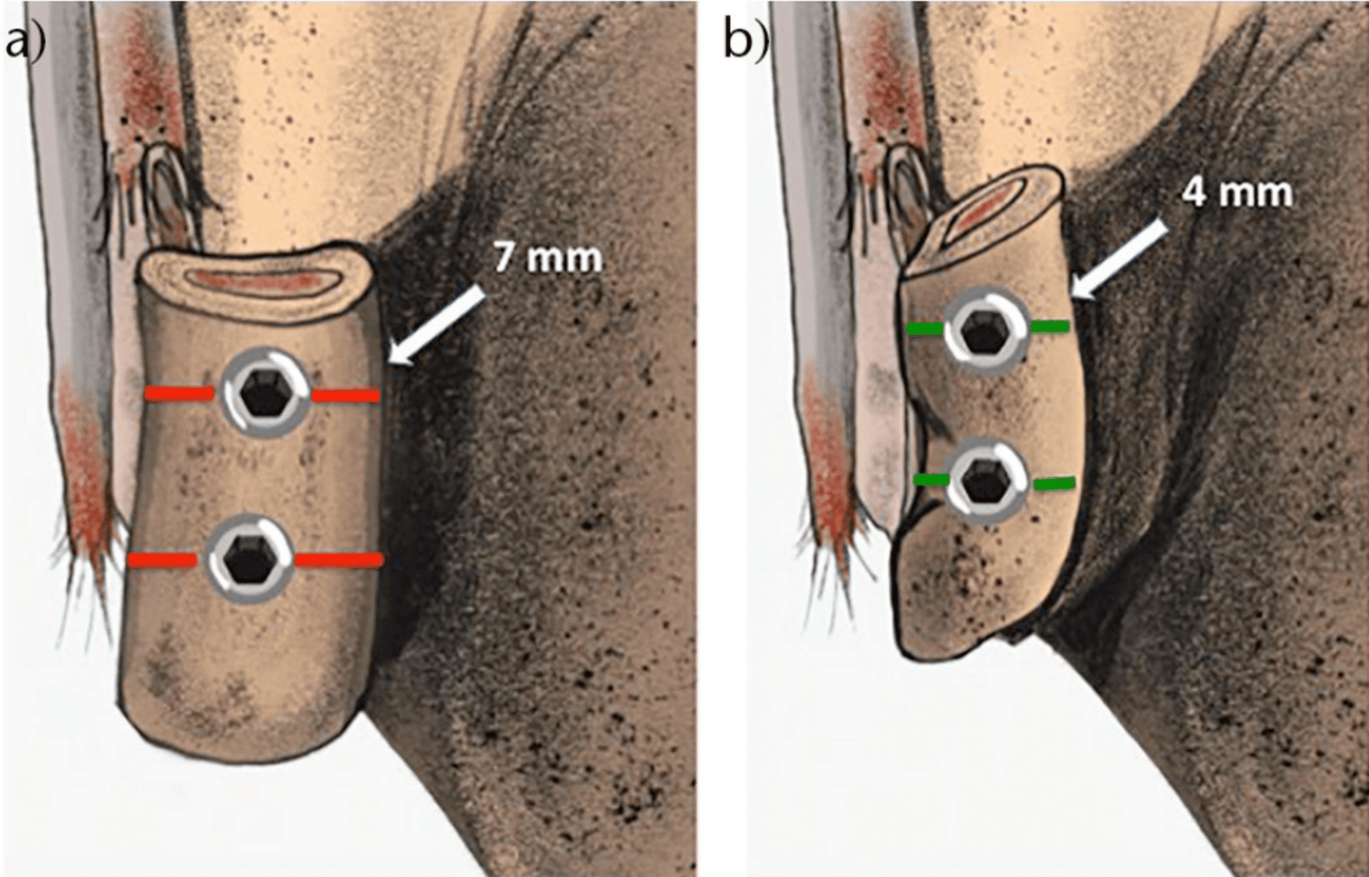
Traditional Latarjet (TL) versus congruent arc Latarjet (CAL) techniques. In the TL technique (a), there is a 7 mm medial and lateral offset on each side of the screw head, while in the CAL (b) technique, the offset is 4 mm in average The figure is taken with permission from [11].

On the contrary, the CAL technique is considered more surgically challenging and less forgiving due to the less bone stock available for screw placement [11,17]. In a radiological study by Dumont et al., it was found that the reserved coracoid bone stock on each side of a 3.2 mm screw was 7 mm when the TL technique was used and 4 mm when the CAL technique was used (Figure 5) [11,17]. This reduced bone stock on either side of the screw increases the risk of fragmentation, especially in patients with smaller coracoids or when larger diameter screws are used for fixation.

Based on the currently available evidence and the higher potential surgical challenges, we routinely perform the TL technique unless the glenoid bone defect is more than 30%, where we may consider performing the CAL technique. 

*Surgical Technique: Coracoid Preparation* 

The coracoid preparation starts by placing a Hohmann retractor on the top of the coracoid. While the arm is still in adduction and internal rotation, the pectoralis minor tendon insertion into the medial aspect of the coracoid body is exposed and released directly from the bone. The arm is then moved into abduction to expose and tighten the coracoacromial ligament. If the surgeon chooses to repair the capsule to the coracoacromial ligament towards the end of the surgery, the release of the coracoacromial ligament should be done 1 cm lateral to its coracoid attachment, otherwise, the coracoacromial ligament can be released directly from the bone. It is advisable to perform all the above releases using the cautery to maintain good homeostasis while ensuring not to extend the release beyond the tip to avoid injuring the blood supply to the coracoid.

A ruler is then used to mark the site of the osteotomy which is 2.5 cm posterior to the tip of the coracoid. The osteotomy can be performed using a self-stopping straight osteotome or an oscillating saw. If the osteotomy is made using the oscillating saw, it is advisable to do 80-90 % of the cut using the saw, and the last 10 - 20% using a straight osteotome to avoid injuring any of the structures inferior to the coracoid. The osteotomy should be made perpendicular to the coracoid. The osteotomized coracoid is then held by a straight Kocher forceps from the side and retracted out of the field while releasing any remaining structures attached, especially on the undersurface of the coracoid using a cautery or a scalpel. 

After the release of all the remnant structures on the medial, lateral, and inferior surfaces of the coracoid, the coracoid is pulled gently out of the wound and flipped to face the inferior surface. Two holes are made in the coracoid using a drill of the same size as screws used to lag the coracoid to the anterior surface of the scapular neck. The two holes should be at least 1 cm apart and in the center of the longitudinal and the medial-lateral axis of the coracoid. It is advisable to place a malleable retractor between the skin and the coracoid while drilling to avoid injury to the skin. The undersurface of the coracoid is then decorticated using a high-speed burr to expose the cancellous bone. The coracoid is then returned to the wound and parked under the pectoralis major muscle (Video 1). 

**Video 1:** Coracoid preparation The video was recorded by the author after securing consent from the patient for taking images and videos for medical and research reasons.

Subscapularis split (SS) vs subscapularis L-shaped tenotomy (ST)

The transfer of the coracoid to the anterior aspect of the scapular neck mandates the exposure of the glenoid and the passage of the coracoid and the attached conjoint tendon through the subscapularis tendon without affecting the integrity of this important anterior shoulder stabilizer. This can be done through two techniques: the partial L-shaped tenotomy of the upper half of the subscapularis tendon (ST) and the subscapularis split (SS). 

Multiple studies have compared the two techniques of glenoid exposure. Although some studies have shown no difference between the two techniques [18], most studies have shown the superiority of the SS technique in various aspects [19-22]. In a relatively large study, Paladini et al. conducted an isometric study comparing the two techniques and found a stronger strength of the subscapularis in the SS group compared to the ST group [19]. In another study by Ersen et al., the SS group had higher subscapularis endurance and better internal rotation durability [20]. In a systematic review and meta-analysis by Davey et al. that included 615 shoulders from five studies, the SS group had significantly better functional outcome measures and lower rates of subscapularis insufficiency [22]. At the structural level, imaging studies showed that subscapularis muscle subjected to a tenotomy had significant fatty infiltration and atrophy while muscle subjected to the splitting technique showed minimal effect [23,24]. Based on the former evidence, it is advisable to perform an SS whenever possible. If the ST technique was adopted, a good repairing technique using nonabsorbable sutures and anchor sutures to minimize the adverse effects associated with this approach must be used. 

Surgical Technique: Glenoid Exposure (Subscapularis Split) 

**Figure 6:**
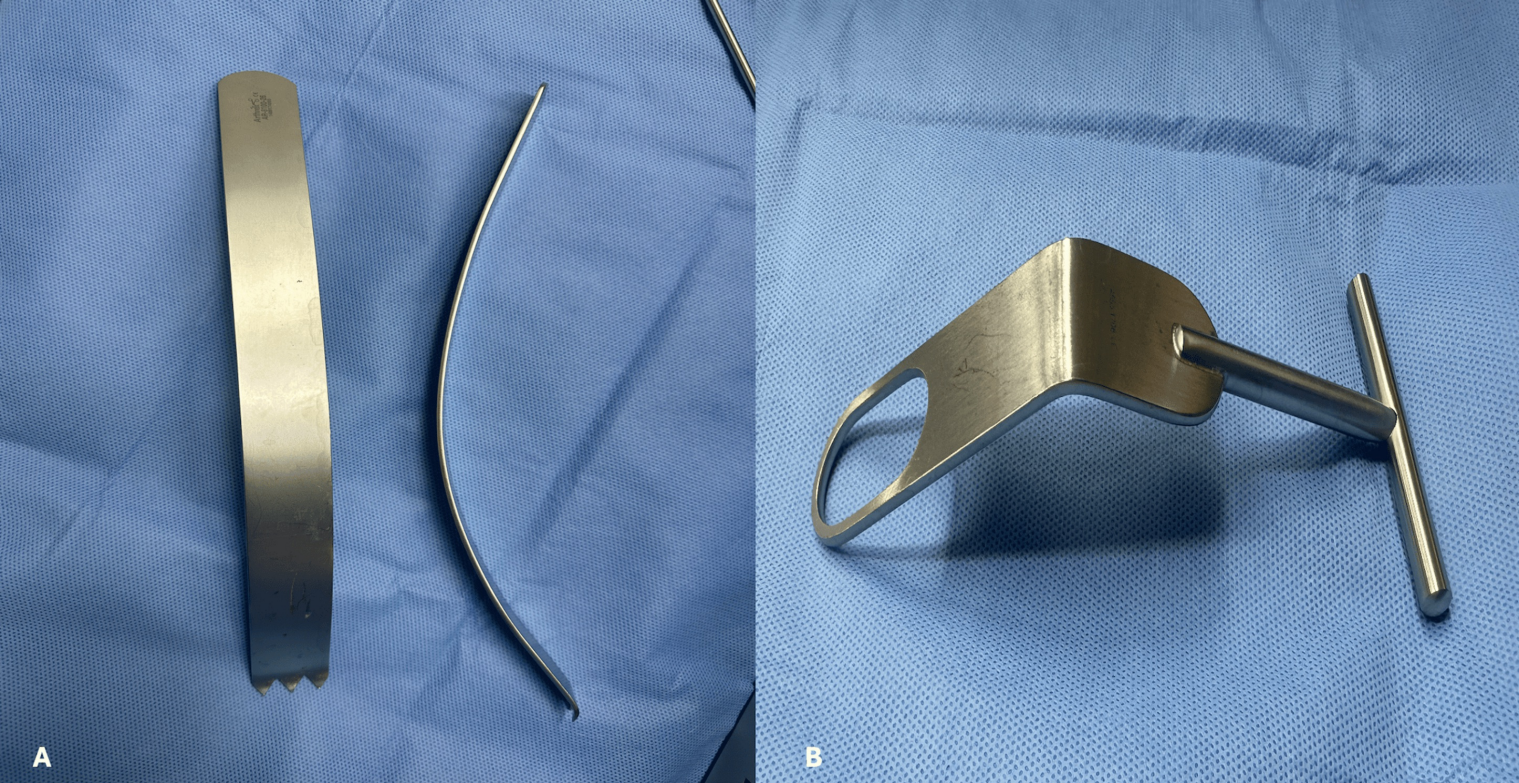
The Kolbel Glenoid retractor (A) and a Fukuda (B) can help in providing good exposure of the anterior glenoid neck and the glenohumeral joint to ensure the proper positioning of the coracoid This picture was taken by the author from the Laterjet set (Arthrex, Naples, FL, USA).

While the arm is in adduction and external rotation to stretch the subscapularis, the upper and lower borders of the subscapularis tendons are identified. The muscle and its tendon are divided in line with the muscle fibers, using cautery or a scalpel, at the junction of the upper two-thirds and the lower one-third. The split is started at the level of the lesser tuberosity and extended medially as needed up to 20 mm medial to the musculotendinous junction, to avoid jeopardizing the nerve supply of the muscle [25]. Mayo scissors can be used to aid in the exposure by placing it in the split and opening it perpendicular to the muscle fibers. A self-retaining retractor is then placed in the split and opened to maintain the exposure. A 4x4 gauze can be placed in the subscapularis fossa to swipe any subscapularis muscle fibers attached to the underlying capsule. After clearly visualizing the capsule, a vertical incision is made in the capsule at the level of the joint line. A small Fukuda retractor is then placed in the capsule split and pulled laterally to allow visualization of the glenohumeral joint. The glenoid neck exposure can be increased by placing a Kolbel Glenoid retractor on the glenoid neck as medially as possible (Figure 6). The labrum is excised between 2 and 5 o’clock position in the right shoulder (7 and 10 o’clock in the left shoulder) using a scalpel or a cautery. The excision of the labrum is further extended medially to include the periosteum over the glenoid neck, at least 2 cm medial to the glenoid rim. A curved osteotome is then used to decorticate the anterior surface of the glenoid neck to bleeding cancellous bone to prepare the surface to which the coracoid will be fixed (Video 2). 

**Video 2:** Glenoid preparation The video was recorded by the author after securing consent from the patient for taking images and videos for medical and research reasons.

Intra-articular versus extra-articular coracoid graft

The classical description of the surgical procedure of the modified Latarjet procedure, otherwise known as the Walch-Boileau technique, involves the fixation of the coracoid to the anterior glenoid neck and the repair of the capsule to the coracoacromial stump resulting in the coracoid being in an intra-articular position [1,4]. This results in the humeral head directly articulating with the coracoid bone, especially in external rotation. This contact is believed to contribute to the increased risk of glenohumeral osteoarthritis, which ranges between 20 to 25% of cases in mid- to long-term studies [26-28]. To minimize the risk of osteoarthritis resulting from this humeral head-coracoid contact, extra-articular placement of the coracoid was proposed. This can be done by repairing the capsule to the native anterior glenoid rim using anchor sutures or passing sutures in tunnels made in the coracoid lateral edge [29]. The first technique is more commonly used because of the risk of graft fragmentation associated with creating tunnels in the coracoid and the tension exerted by the capsule on the body of the coracoid. A minimal number of studies reported on the incidence of osteoarthritis after extra-articular coracoid placement. In a study by Bouju et al, the reported incidence was 5.2% at 13-year follow-up [30]. Boileau et al. reported a 9% incidence of osteoarthritis in a 33-month follow-up period in 70 shoulders after the arthroscopic Latarjet procedure with extra-articular graft placement [31]. 

Apart from the site of placement of the coracoid, there are other risk factors of osteoarthritis post-shoulder dislocation including older age at the onset of instability, recurrent dislocations, glenoid fracture, excessive screws obliquity, and lateral overhanging of the coracoid graft beyond the glenoid rim [29]. Another important variable to consider is the natural progression to glenohumeral osteoarthritis after at least one shoulder dislocation. In a cross-sectional study by Kruckeberg et al. on 154 patients aged less than 40 years with a mean follow-up of more than 15 years, the incidence of symptomatic osteoarthritis was 22.7% [32]. In another study by Hovelius et al., a prospective multicenter center study was conducted on 229 shoulders with a mean follow-up of 25 years. Glenohumeral osteoarthritis was moderate to severe in 18% of patients after a single dislocation incident and 39% in patients with recurrent dislocations [33]. This indicates that there are multiple other variables that need to be considered when studying the relationship between the site of coracoid placement and the risk of osteoarthritis. 

On the contrary, studies have shown that extra-articular coracoid placement is associated with a decrease in the arc of motion especially external rotation when compared to intra-articular coracoid placement. In a cadaveric study by Itoigawa et al. on 18 fresh-frozen shoulders, the external rotation was significantly lower in the extra-articular group at 0 and 60 degrees of abduction but not at the end range of motion [34]. 

Recently, more studies started questioning the importance of repairing the capsule and its role as one of the factors contributing to the stability of the shoulder after performing a Latarjet procedure. In the cadaveric study by Yamamoto et al., the role of the capsule as a stabilizer was limited to 23-24% at the end range of motion with no contribution in mid-range [23]. Although the capsule is elongated when repaired to the remnant of the CAL due to the coracoid thickness and the additional length of the CAL, some studies have reported a decrease in external rotation because of this repair [29,35,36,37]. In a prospective cohort study by Sahu, 30 patients who received a Latarjet procedure with capsular repair were compared to 31 patients who had a Latarjet procedure without capsular repair. The capsular repaired group had a significantly larger external rotation deficit compared to the normal contralateral shoulder at 90 degrees of shoulder abduction but not when adducted. This deficit in external rotation was not significant in any shoulder position in the group that had no repair. Furthermore, there was no clinically significant difference in various functional outcome used between the two groups [37]. In another study by Ranalleta et al., the functional outcomes of 68 athletes treated with a modified Latarjet procedure without capsular repair were studied. All surgeries were revision surgeries after a previously failed shoulder stabilization procedure. The authors reported excellent functional outcomes with most athletes returning to sports at the same competitive level [38]. 

To our knowledge, no single clinical study that compares the effect of intra-articular versus extra-articular coracoid placement on the arc of motion, the risk of glenohumeral osteoarthritis, and the risk of re-dislocation, while taking into consideration all other variables exists. The same applies to a clinical comparison between capsular repair versus no repair. In our center, we do not perform any form of capsule repair leaving the coracoid in an intra-articular position. Based on our observations from our patients, we lean more toward the idea that repairing the capsule may restrict the arc of motion and increase the operative time without any potential advantageous improvement in functional and clinical outcomes.

Surgical Technique: Coracoid Fixation

Multiple methods of coracoid fixation have been used in the literature including 2.8 mm hole buttons, cerclage sutures, 3.5 mm screws, 3.75 mm cannulated screws, and 4.5 mm malleolar screws. Apart from the surgeon's preference, multiple factors should be considered when choosing the appropriate method of fixation. First, the implant used should provide adequate fixation and sufficient compression to facilitate the consolidation of the coracoid to the anterior glenoid neck. Second, the surgeon should consider using a modality that provides a two-point fixation for rotational stability. Third, the superior-inferior and medial lateral measurements of the coracoid should be carefully considered to avoid fragmentation of the coracoid or the creation of stress risers between the holes made in the body of the coracoid. Technically, the holes made in the coracoid should at least be 1 cm apart in the superior-inferior axis and should be centered in the medial-lateral axis. The “safe zone” was identified by Boutsiadis et al. as the distance between the implant and the coracoid osteotomy which should be greater than the diameter of the implant used [39]. Another important technical consideration is the medial and lateral offset, which is the distance from the implant to the medial and lateral coracoid edge respectively. The surgeon should carefully try to increase this offset by choosing a smaller implant but without jeopardizing the adequacy of the fixation. In our center, we mostly use two 3.75 mm cannulated screws unless technically not possible. The use of 3.75 mm screws provides an adequate safe zone in the superior-inferior axis and a safe medial and lateral offset in most coracoids. Furthermore, the use of Kirschner wires (K-wires) before drilling the anterior glenoid neck ensures the parallelism of the screws and avoids hyper-divergence or drilling into the glenohumeral joint. 

After the excision of the labrum between 2 and 5 o’clock position in the right shoulder (7 and 10 o’clock in the left shoulder) and the elevation of the periosteal flap with decortication of the anterior glenoid neck to accommodate the coracoid graft, fixation of the coracoid is done. The coracoid is held using a thin bone holding forceps or parallel drill guide. The coracoid is placed between 3 and 5 o’clock position in the right shoulder (7 and 9 o’clock in the left shoulder) on the anterior glenoid neck. While still holding the coracoid in the desired position on the anterior glenoid neck, two K-wires are inserted through the holes previously created in the coracoid. The K-wires inserted should be as parallel to each other and perpendicular to the glenoid neck, avoiding more than 10-degree divergence between the 2 K-wires or medial and lateral divergence. After the insertion of the K-wires, the coracoid holding device is removed, the coracoid is pushed to the anterior glenoid rim using a cobb, and its position should be checked by direct visualization and palpation with the tip of a long pickup, ensuring the lateral edge of the coracoid is flushed to the anterior glenoid rim. If the coracoid is medialized or overhanging the glenoid rim, one or two of the K-wires should be removed and then reinserted after adjusting the coracoid to the desired position. If the lateral edge of the coracoid is not flat, a rongeur or a burr can be used to flatten it. The K-wires inserted are stopped just before going through the posterior glenoid neck cortex. The length of the screws to be used is measured using another K-wire of the same length, a special measuring device in the set used, or a depth gauge. 

A cannulated drill is then used to drill the anterior glenoid neck cortex only. It is advised to push the K-wires through the posterior cortex of the glenoid after the measurement and before drilling to avoid pulling out the K-wires while drilling. 

After drilling the near anterior glenoid cortex, two cannulated screws are inserted over the K-wires. It is advised to start with the lower screw first, using a partially threaded one followed by a fully threaded screw in the upper hole. The screws should be tightened sequentially using a two-finger tightening technique to avoid fragmentation of the coracoid. The lower partially threaded screw will act as a compressing screw, and the fully threaded upper screw will act as a holding screw. The final position of the coracoid is checked for the last time before pulling out the K-wires. If the capsule repair is adopted, absorbable sutures are used to repair the capsule to the coracoacromial ligament stump for intra-articular positioning of the coracoid, or threaded anchor sutures inserted to the anterior glenoid rim if an extra-articular position is desired. It is important to repair the capsule while the arm is in maximum external rotation and adduction (Video 3).

**Video 3:** Coracoid fixation The video was recorded by the author after securing consent from the patient for taking images and videos for medical and research reasons.

Irrigation of the wound is done using copious amounts of sterile normal saline. All retractors inserted to open the SS and the deltopectoral interval are removed. The SS is not sutured to avoid tightening of the subscapularis. The lowered two-thirds of the deltopectoral interval is sutured using a running technique, to avoid injuring the cephalic vein. The subcutaneous layer is then closed using interrupted absorbable sutures followed by the skin closure.

## Conclusions

Multiple variations were proposed in the literature for the modified Latarjet procedure, otherwise known as the Walch-Boileau procedure. The current evidence is based on a group of low-evidence heterogenous studies due to the absence of well-controlled clinical studies comparing multiple aspects of these variations. Based on the currently available evidence, it can be concluded that both arthroscopic and open Lartarjet procedures yield excellent functional outcome results with low complication and recurrence rates, but a much steeper learning curve is associated with the arthroscopic procedure. Both TL and CAL procedures are effective in the management of recurrent shoulder dislocation with glenoid bone loss defects of less than 30%. In the CAL procedure, the medial border of the coracoid is fixed to the anterior glenoid neck yielding a larger non-beneficial bone block effect and theoretically unproven lower risk of osteoarthritis but a higher risk of coracoid fracture and fragmentation. Subscapularis tenotomy is superior to the L-shaped subscapularis tenotomy in yielding better function outcomes with lower rates of subscapularis insufficiency. Extra-articular positioning results in a theoretical advantage of lowering the risk of glenohumeral osteoarthritis but may be associated with the limitation of external rotation. Recent clinical studies show no clinical superiority in the functional outcome after Latarjet in patients who received a capsular repair.
